# A methodological and clinical approach to measured energy expenditure in the critically ill pediatric patient

**DOI:** 10.3389/fped.2022.1027358

**Published:** 2022-10-24

**Authors:** Jaime Silva-Gburek, Paola Hong Zhu, Marwa Mansour, David Walding, Jorge A. Coss-Bu

**Affiliations:** ^1^ Pediatric Critical Care Medicine, Children’s Mercy Hospital, University of Missouri-Kansas City School of Medicine, Kansas City, MO, United States; ^2^ Division of Critical Care, Department of Pediatrics, Baylor College of Medicine, Houston, TX, United States; ^3^ Texas Children’s Hospital, Houston, TX, United States; ^4^ Department of Biomedical Engineering, Texas Children's Hospital, Houston, TX, United States

**Keywords:** basal metabolism, indirect calorimetry, critically ill, oxygen consumption, carbon dioxide production, substrate oxidation, respiratory coefficient, pediatrics

## Abstract

The metabolic response to injury and stress is characterized initially by a decreased energy expenditure (Ebb phase) followed by an increased metabolic expenditure (Flow phase). Indirect calorimetry is a methodology utilized to measure energy expenditure and substrate utilization by measuring gas exchange in exhaled air and urinary nitrogen. The use of indirect calorimetry in critically ill patients requires precise equipment to obtain accurate measurements. The most recent guidelines suggested that measured energy expenditure by indirect calorimetry be used to determine energy requirements. This article reviews the methodological and clinical use of indirect calorimetry in critically ill pediatric patients.

## Introduction

The metabolic response to injury and stress, from a critical illness, is represented by systemic manifestations. The initial response after an injury is characterized by a decrease in metabolic expenditure (Ebb phase) during the first 2–3 days, this response is intended to preserve energy; it is immediately followed by a flow phase, where there is a considerable increase in metabolic expenditure and a catabolic response that is proportional to the initial injury (1–9). The measurement of metabolic expenditure and the changes in the oxidation of carbohydrates, proteins, and lipids in a critically ill patient can be obtained by using the indirect calorimetry method (10–13).

## Methodology for the measurement of metabolic expenditure

### Indirect calorimetry: Gas exchange method

Indirect calorimetry (IC) is a method used to measure metabolic expenditure and substrate utilization through the measurement of gas exchange of exhaled air and urinary nitrogen. This method is based on 3 premises: (1) inspired oxygen (O_2_) is used in its totality to oxidize different substrates, (2) exhaled carbon dioxide (CO_2_) is a product of the complete combustion of these substrates, and, (3) non-protein nitrogen deposed in urine is a product of free amino acids oxidation (14–18).

The gas exchange methodology measures oxygen consumption (VO_2_) and carbon dioxide production (VCO_2_) by quantifying the differences of O_2_ and CO_2_ between inspired and exhaled gas. The following formulas include the Haldane transformation:

$$\begin{array}{r} VO_{2}(\mathrm{ml}/\min)=\;\{\left[\left(1-\mathrm{FE}O_{2}-\mathrm{FEC}O_{2}(1-\mathrm{FI}O_{2}\right)\right]\times (\mathrm{FI}O_{2}]-\mathrm{FE}O_{2})\}\times \mathrm{VE} \end{array}$$

FEO_2_ is the concentration of expired O_2_, FIO_2_ is the concentration of inspired oxygen, FECO_2_ is the concentration of expired CO_2_, and VE is the expired volume of air over time in milliliters per minute (ml/min).


$$\mathrm{VC}O_{2}\left(\mathrm{ml}/\min\right)=\mathrm{VE}\left(\mathrm{FEC}O_{2}\right)$$

The values for VO_2_ and VCO_2_ are converted into a caloric equivalent of resting metabolic rate (RMR) using formulas created by Weir (18):

$$\mathrm{RMR}\left(\mathrm{Kcal}/\mathrm{kg}/\mathrm{day}\right)=[VO_{2}\left(3.941\right)+\mathrm{VC}O_{2}\left(1.11\right)]\times 1.440$$

The RMR is adjusted by taking into consideration the protein metabolism which is quantified by using the total urinary nitrogen (TUN) level over 24 h (19, 20):

CorrectedRMR=RMR−2.17×TUN(g/day)

These values are used to calculate the oxidation indexes of different substrates (carbohydrates, protein, and lipids) by using Conzolazio's constants; the TUN level is measured over 24 h (21). The measured gas exchange represents the composition of oxidized substrates, 1 kcal requires 0.236, 0.214, and 0.199 L of oxygen to oxidize protein, carbohydrates, and lipids respectively. Similarly, 1 kcal produces 0.190, 0.151, and 0.199 L of carbon dioxide from the oxidation of protein, carbohydrates, and lipids respectively. When oxidative reactions occur in the human body, energy is released in corresponding units of adenosine triphosphate (ATP). For every 1 mol of oxidized glucose, palmitate, and amino acids, 3.00, 2.85, and 2.25 units of ATP are produced respectively. These values help explain a fundamental concept of physiology: the most efficient way to utilize oxygen in the production of energy (adenosine triphosphate) is *via* the oxidation of glucose; the oxidation of lipids and protein requires higher levels of oxygen. It is important to clarify that by simultaneously using IC and TUN, it is possible to calculate a “net” oxidative index of carbohydrates, proteins, and lipids (19–23). Nitrogen excretion in the urine measured as urea nitrogen has been shown to be highly variable in critically ill adults and children (23–26), therefore, it is recommended to measure total urinary nitrogen by the Kjeldahl method to obtain accurate measurements and minimize variability (20, 22, 27).

The VCO_2_/VO_2_ relationship represents the respiratory quotient (RQ) and its value is constant and unique for each substrate, it is important to point out that the RQ does not represent a change in the absolute values of VO_2_ and VCO_2_, which are indices that impact the cardiopulmonary function. The corresponding RQ values for the net oxidation of carbohydrates, protein, and lipids are 1.00, 0.80, and 0.70 respectively. The RQ index that does not involve protein contribution (npRQ) has a value of 0.70–1.0, where values >1.0 indicate the synthesis of fat from glucose substrate, a process called lipogenesis. Several clinical trials in patients admitted to the pediatric critical care unit (PICU) have reported that fluctuations in metabolism or an excessive glucose caloric intake, may alter npQR values (22, 28–32).


$$\mathrm{npRQ}=\left[\mathrm{VC}O_{2}-4.8\;\left(\mathrm{TUN}\right)\right]/\left[VO_{2}-5.9\;\left(\mathrm{TUN}\right)\right]$$

The relative percentage of metabolized substrates is obtained using the following formulas:

(1) Carbohydrates (%): [(carbohydrates + lipids)/corrected RMR] × [(97.4 npRQ—68.9)/(59.4 npRQ—30.8)]
(2) Lipids (%): [(carbohydrates + lipids)/corrected RMR] × [[38.1 (1-npRQ)]/(59.4 npRQ—30.8)]
(3) Proteins (%): Protein/corrected RMR. Protein = 6.25 × 4.2 × UUN (g/day).

Carbohydrates+lipids=correctedRMR−Protein

## Methodology for the quantification of metabolic expenditure

Metabolic expenditure calculations may be obtained by using either an open or closed system.

### Open circuit method

The patient is connected to a spirometry system with 100% oxygen *via* a mouth adaptor, facial mask, or endotracheal tube, which is then connected to a kymograph. During the patient's inspiration and exhalation, oxygen is consumed, and carbon dioxide is produced. The carbon dioxide and water vapor are absorbed by the system, therefore, the changes in volume detected by the spirometer represent the consumption of oxygen by the lungs of the subject. This technique is not free of errors, and these may result in incorrect metabolic measurements. Another limitation of this system is its short measurement time (generally <10 min), which is not long enough for oxygen and carbon dioxide deposits to stabilize in the human body. Additionally, the metabolic consequences of breathing 100% oxygen on carbon dioxide absorption *via* soda lime have not been well documented. Currently is no longer used in critically ill patients.

### Closed circuit method

This method has been used for the continuous quantification of metabolic expenditure in both subjects with spontaneous respiration and mechanical ventilation. During the measurement, the patient's head is positioned in a transparent plastic chamber with no leaks (canopy) and breathing room air at a constant flow. As the patient exhales, gas is collected in a mixing chamber, and the values of oxygen and carbon dioxide are quantified. The subject's VO_2_ and VCO_2_ are calculated by multiplying the airflow in ml/min (room air at a 0.21 oxygen concentration) by the absolute values of the differences in concentrations between O_2_ and CO_2_ in inspired air minus exhaled air. A key factor to consider when using this method is that higher airflow will decrease the differences of O_2_ between inhaled and exhaled air, therefore the equipment used to analyze O_2_ and CO_2_ levels must have the appropriate sensitivity to detect small differences in exhaled and inhaled air.

## Using indirect calorimetry during mechanical ventilation

**Figure 1:**
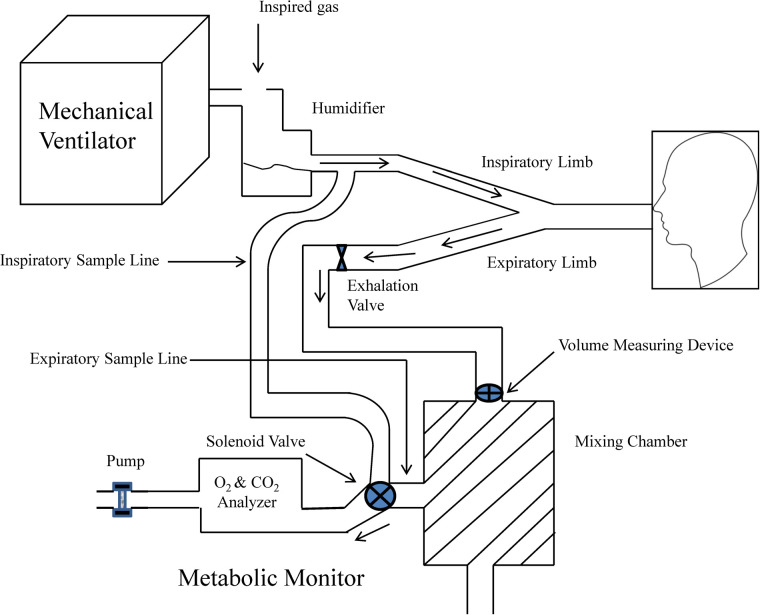
Graphic representation of metabolic equipment with mixing-chamber and its interface with mechanical ventilation. Modified from Weissman and Kemper (33).

**Figure 2:**
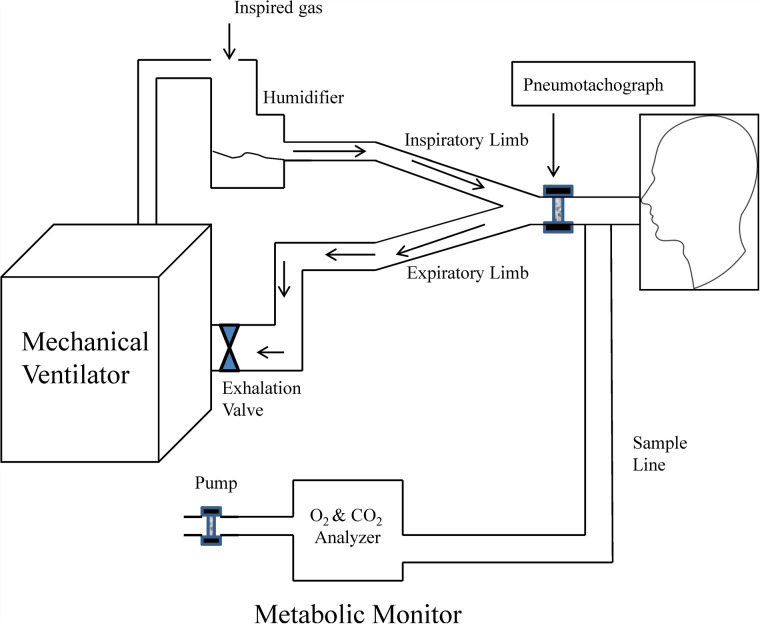
Graphic representation of metabolic equipment with breath-by-breath analysis and its interface with mechanical ventilation. Modified from Weissman and Kemper (33).

The use of IC for the measurement of VO_2_ and VCO_2_ during mechanical ventilation requires of a full understanding of the mechanical ventilator and metabolic equipment being utilized (33). The most important components of the metabolic system are the volume measurement devices (pneumotachograph, turbine, hot wire, ultrasonic, etc.) and the gas analyzers for oxygen and carbon dioxide (paramagnetic, zirconia, fuel cell, mass spectrometry, etc.); therefore, metabolic systems must-have components of high precision to obtain acceptable measurements. The current metabolic systems include precise gas analyzers with faster response time (zirconia, fuel cell, paramagnetic, etc.) and advanced electronic components; this has allowed to improve the precision and facilitate the process to obtain these measurements. The two most used systems to measure metabolic expenditure in patients receiving mechanical ventilation are (1) Mixing-chamber (Figure 1) and (2) Breath-by-breath technique (Figure 2), the last system requires fast response gas analyzers and high precision volume measurement devices. The main features that any metabolic system should have to measure metabolic expenditure in patients on mechanical ventilation are listed in Table 1.

**Table 1:** Characteristics of the metabolic system used for the measurement of energy expenditure during mechanical ventilation.

1.	There must be no leaks in the system
2.	The system must be stable and have the capacity to reach a steady state
3.	The source of inspired oxygen must be stable
4.	The O_2_ and CO_2_ analyzers must be precise and tolerate high levels of inspired oxygen
5.	High levels of PEEP will affect the precision of the O_2_ and CO_2_ analyzers.
6.	The devices that measure volume must be precise within a range of volume (50–1.500 ml) and flows (1–100 ml/min)
7.	The calculation of VO_2_ and VCO_2_ must account for water vapor pressure.
8.	The values of VO_2_ and VCO_2_ should correspond with the time of the values for the concentration of gases and the measurement of volumes.
9.	Gases are modified by variations in temperature, pressure, and humidity and their values should be expressed in STPD units
10.	Describe the characteristics and limitations of the metabolic cart

O_2_, oxygen; CO_2_, carbon dioxide; PEEP, positive end-expiratory pressure; VO_2_, oxygen consumption; VCO_2_, carbon dioxide production; STPD, standard temperature (0°C), pressure (760 mmHg), and dry.

The appropriate interpretation of the measured parameters of IC requires an understanding of the premises and technical aspects of this methodology. The continued advancement of technology and the manufacturing of highly precise portable metabolic carts have made the use of indirect calorimetry in critically ill patients a reality, this includes subjects on mechanical ventilation. There are several factors that can make the real-world application and interpretation of this methodology difficult in an intensive care unit, among these are: (1) the calculation model, (2) calorimetric factors, (3) leak around the endotracheal tube, (4) fraction of inspired oxygen above 0.60, (5) elevated levels of positive end-expiratory pressure (PEEP), generally >10 cm H_2_O, (6) limited stability of gas analyzers, (7) inability to reach a steady state, and (8) human factors. The duration of the indirect calorimetry study is extremely important when measuring metabolic expenditure to reach a steady state, this is defined as a variation coefficient [(standard deviation/average) × 100] less than 10% which represents an equivalent to energy expenditure over 24 h. Clinical studies in critically ill adults and children have found that a shorter duration of indirect calorimetry measurements ranging from 3 to 5 min is adequate to reach a steady state with precise findings, from a clinical standpoint; other authors have found that indirect calorimetry measurements lasting 30 min may correspond to 24-h studies.

The total energy expenditure (TEE) in healthy subjects can be divided into three components: basal energy expenditure (BEE), thermogenesis, and physical work. The BEE represents 60%–70% of the TEE, thermogenesis equals 10% and physical work corresponds to 20%–30% of the TEE. During times of stress or injury, the values of these components can change in relation to the degree of injury, diet, and physical activity. Diet-induced thermogenesis (DIT) is defined as the amount of energy required to absorb, process and store nutrients and is equal to the increase in energy expenditure as it relates to the post-absorption state. Doubling the intake of calories compared to the REE during the acute phase of disease produces a 10%–20% increase in the DIT, with an increase in VO_2_ and VCO_2_ that will be reflected in the increased ventilatory support and stress on the cardiovascular system in the critically ill patient (27, 32, 34–39); which is why the current recommendations are to not administer an excess of calories compared to REE during the acute disease phase or immediate post-operative phase unless an indirect calorimetry study has been performed (3, 32, 38–41).

The possible benefits of using indirect calorimetry in critically ill patients are (1) quantification of energy expenditure in patients unresponsive to medical management whose nutritional plan is based solely on estimated energy requirement formulas, (2) measurement of energy expenditure in critically ill patients with organ failure and prolonged use of parenteral nutrition, (3) to evaluate the effects of parenteral nutrition on the respiratory system of patients with acute and chronic respiratory failure on ventilatory support, and (4) to measure the consumption of oxygen during the weaning phase of respiratory support. An article by Mehta et al. (32), described indications for the measurement of resting energy expenditure (REE) in the pediatric intensive care unit: these recommendations are incorporated into the clinical guidelines for nutritional support of the critically ill pediatric patient, published by the American Society of Parenteral and Enteral Nutrition (A.S.P.E.N.) (39, 41). In conclusion, indirect calorimetry allows us to understand the process of energy utilization during periods of acute stress and injury, although this has not been shown to improve outcomes in clinical studies. More research is needed to analyze the benefits of indirect calorimetry, its uses in calculating energy requirements, preventing overfeeding or underfeeding, and its cost-benefit relationship in the critical care unit.

## Other methods to quantify energy expenditure

### Stable isotopes

Procedures used to measure energy expenditure in humans include indirect and direct calorimetry, stables isotopes, 24-h cardiac frequency monitoring, and the use of monitors to measure physical activity. Direct calorimetry quantifies the dissipation of heat during a state of rest, where the heat that is lost is equal to the energy that is produced. Indirect calorimetry quantifies heat produced based on gas exchange and calculated energy expenditure at rest. Total energy expenditure (TEE) considers physical activity and can be measured by using doubly labeled water. This method uses stable isotopes (^2^H_2_O, H_2_^18^O, NaH^13^CO_3_) to quantify energy expenditure (42–46).

The method for using doubly labeled water with stable isotopes was developed 50 years ago and is based on the differences in the production of ^2^H_2_O and H_2_^18^O in the fluid compartment of the human body (47–49). After a period of equilibrium, ^2^H and ^18^O dissolve in this fluid compartment, while ^18^O is eliminated *via* respiration in the form of carbon dioxide. The VCO_2_ is calculated by subtracting the difference in the production of both isotopes. By using a respiratory coefficient (VCO_2_/VO_2_) of 0.85, the metabolic expenditure is estimated by using the values for VO_2_ and VCO_2_. This doubly labeled water technique has been validated against indirect calorimetry and is now considered the gold standard for measuring TEE in ambulatory subjects. The sources of error using this method are due to analytical errors of the mass spectrophotometer when measuring the percentage of enrichment of the isotopes, biological variability in the enrichment of the isotopes, division of the isotope during the production of carbon dioxide and water vapor, total body water measurement and the use of an estimated or calculated 24-h respiratory coefficient. It is impossible to implement this technique with patients in the critical care unit given their constant changes in fluid over various body compartments, hence it is reserved for outpatient measurements.

Previously, the production of carbon dioxide during respiration has served as an index for substrate oxidation and energy expenditure. The isotopic dilution technique consists of the incorporation and dilution of the ^13^C marker in bicarbonate which is marked with the isotope NaH^13^CO_3_ (50–55). The quantification of the degree of dilution of the isotope in the exhaled air or in the blood is used to calculate the level of carbon dioxide production. Energy expenditure calculation requires the quantity of released energy per liter of carbon dioxide production or the energy equivalents of CO_2_ (Eq E CO_2_) that reflect the coefficient value of the dietary intake (56). Two fundamental factors that may affect this technique are (1) errors in the measurement of labeled bicarbonate and (2) the possibility that labeled bicarbonate may not dissolve with the CO_2_ produced in the mitochondria. Requiring a precise knowledge of the bicarbonate correction factors necessary for physiologic function (fractional recovery) is another limitation. Several clinical studies have estimated fractional recovery of premature neonates and pediatric patients in critical care between 0.63 and 0.95. It is necessary to emphasize the importance of quantifying fractional recovery values of bicarbonate according to the specific populations being studied, without solely relying on values obtained in adults. In conclusion, isotopic methods and indirect calorimetry are considered complementary methods since the former requires VCO_2_ which is measured using the latter.

### Fick method

In patients with thermodilution catheters in the pulmonary artery, we can measure VO_2_ and VCO_2_ through the measurement of cardiac output (CO) and differences in arterial and venous oxygen content and carbon dioxide by using the Fick method. The Fick method is based on the formula: VO_2_ Fick = CO × [(SaO_2_ – SvO_2_) × Hb × 0.0139 + (PaO_2_ – PVO_2_)× 0.003], CO is cardiac output (L/min); Hb is the value of hemoglobin (g/L); SatO_2_ and SVO_2_ correspond to oxygen saturation (%) of arterial and mixed venous blood; PaO_2_ and PVO_2_ represent partial pressure of oxygen (torr) in arterial and mixed venous blood, respectively. The problems related to a precise measurement using the Fick Method regarding the quantification of VO_2_, include: (1) values lower than the total oxygen consumption since this method does not take into consideration the VO_2_ of bronchial and Thebesian veins, (2) it may not show changes in VO_2_ during the initial stages of injury, and (3) the formulas for calculation of oxygen consumption and oxygen transport are mathematically coupled.

Some research studies have concluded that, compared to the Fick Method, indirect calorimetry is the non-invasive method of choice for measuring VO_2_ in adults. Nowadays, the use of the Fick Method in critically ill children is limited since there are few indications for the placement of pulmonary artery thermodilution catheters and their insertion is technically difficult in small children.

## Methods for calculating caloric needs

Critically ill pediatric patients admitted to an ICU have different caloric needs when compared to healthy children; in relation to their metabolism, growth index, comorbidities, and preexisting energy reserves; hence it is difficult to evaluate the metabolic requirements of this patient population. Therefore, the serial monitoring of their metabolic energy expenditure during the course of the disease is of utmost importance. When you do not have access to the specific equipment required to measure energy expenditure in a pediatric patient in the ICU you may cautiously use reference values to estimate their caloric needs.

### Reference values for individuals less than 18 years old

The reference values commonly recommended for estimating basal energy expenditure in children and adolescents include:

1. Harris-Benedict Equation: (16) published in 1919 it is one of the most commonly used equations to estimate basal energy expenditure; it was derived from measurements performed on 94 newborns and 239 individuals (136 men and 103 women) older than 16 years of age. This equation has never been validated in pediatric patients.
2. Formulas published by the Food and Agriculture Organization of the United Nations (FAO), and the World Health Organization (WHO) of the United Nations (57); these formulas were used in clinical studies including 6,100 participants.
3. Talbot published his equation for calculating basal energy expenditure in 1938, based on measurements performed on 3,000 pediatric subjects; 2,220 females and 800 males (17).
4. Schofield Equations (58); are based on the results of the clinical study performed by the FAO and WHO. It's important to mention that, due to the variability of the metabolic state of critically ill children during their ICU stay, these reference values may result in an overestimation or underestimation of the patient's actual energy requirements. This was demonstrated by Kyle et al. (59), by showing that in a group of 240 critically ill children admitted to an ICU for longer than 48 h, a negative metabolic balance during the first 8 days of the ICU stay was obtained by using the values from the Schofield Equation.

### Correction factors

With the use of correction factors for stress and activity, in addition to basal energy expenditure equations, there have been published evaluations overestimating or underestimating the metabolic needs of critically ill patients. This highlights the need for the use of indirect calorimetry as a tool for the quantification of these metabolic needs in this patient population (2, 3, 5, 11, 15, 23, 28, 30, 38, 39, 60–66).

Coss-Bu et al., evaluated the basal energy expenditure values measured by indirect calorimetry in 55 children on mechanical ventilation; the corresponding values using the Harris-Benedict and Talbot equations of each patient were corrected with two factors of 1.3 and 1.5 (2). The corrected basal energy expenditure results obtained were compared to the resting energy expenditure measured by indirect calorimetry and found to have vast differences, thus concluding that indirect calorimetry is the only precise method to measure the metabolic requirements of critically ill pediatric patients.

### Predictive equations

It's been shown that the use of formulas or predictive equations is more appropriate than the use of reference values, these equations have been developed using multiple linear regression models that include different variables (gender, body weight, height, body temperature, heart rate, inotropic infusion rate, diagnosis of sepsis, ICU length of stay, mechanical ventilation, etc.), performed in both adults and pediatric patients admitted to an ICU (67–71). Although, these predictive formulas should be used with caution given the variability of the metabolic state of these critically ill patients during their hospital stay.

### Use of carbon dioxide production to estimate energy expenditure

Recent studies in critically ill adults and children have reported on a simplified formula to calculate basal energy expenditure based solely on the measurement of the production of carbon dioxide or a derivation of different values of the respiratory coefficient (0.80, 0.85, and 0.89) (72–76).


$$\mathrm{RMRvc}o_{2}\left(\mathrm{Kcal}/\mathrm{kg}/\mathrm{day}\right)=5.534\times \mathrm{VC}O_{2}\times 1440$$

These calculations have been made possible thanks to the measurement of carbon dioxide concentrations on mechanical ventilators currently used in intensive care units. VCO_2_ es calculated by taking into consideration minute ventilation and the concentration of exhaled carbon dioxide. The consensus that several authors reached was that the measurement of basal energy expenditure based solely on VCO_2_ in the absence of VO_2_ measurements is simple and attractive and could be used in lieu of predictive equations, although indirect calorimetry, which includes both the measurement of VCO_2_ and VO_2_, remains the recommended form of calculating energy expenditure in critically ill patients on mechanical ventilation.

## Use of indirect calorimetry in critically ill children

### Targeted indirect calorimetry

Basal energy expenditure measured by indirect calorimetry is the gold standard for the measurement of metabolic needs in critically ill patients. This recommendation is included in the most recent pediatric nutritional clinical guidelines of A.S.P.E.N., S.C.C.M. (41), and E.S.P.E.N. (77), which propose obtaining indirect calorimetry calculations in patients with fluctuating metabolic alterations or who have evidence of malnutrition. These guidelines highlight the importance of identifying imbalances between caloric intake and measured energy expenditure with the goal of avoiding an excessive or inadequate nutritional consumption and this way preventing any adverse consequences that this might entail. Given the ever-changing nature of metabolic conditions that are reflected in a patient's energy requirements through the course of their disease, the calculation of their resting energy expenditure should be obtained serially, and corresponding changes must be incorporated to their caloric intake.

The current clinical nutrition guidelines for critically ill pediatric patients that were recently published by A.S.P.E.N. and S.C.C.M. state that indirect calorimetry plays a vital role in patients admitted to the PICU whose metabolic needs are constantly changing, whether they be hypermetabolic or hypometabolic. In a clinical study by Metha et al. (32), in critically ill pediatric patients admitted to a PICU it was found that indirect calorimetry in high-risk patients helped prevent accumulated excesses and deficits in metabolic balance. Kyle et al. (40), published a prospective observational study that included 150 critically ill children and was meant to determine how many of them met the criteria for indirect calorimetry measurements during their first week of IC stay. The results showed that 75% of the enrolled patients were candidates for the use of indirect calorimetry and that one-third of these patients had two or more criteria for its use. With this study, the authors highlighted even more the importance of performing multi-center clinical studies to establish factors that determine the cost-benefit ratio of the use of indirect calorimetry in patients admitted to a critical care unit.

### Measuring energy expenditure in children with congenital heart disease

Several authors reported measurements of energy expenditure using indirect calorimetry in children after correction for congenital heart disease (CHD) (6, 78–81). De Wit et al. (78), reported energy expenditure measurements in 21 mechanically ventilated children, the children who underwent cardiopulmonary bypass (CPB) had REE of 73.6 ± 14.45 vs. 58.3 ± 10.29 kcal/kg/day in children who underwent non-bypass surgery, suggesting that CPB exposure increased energy expenditure and the need to increase caloric requirements postoperatively. Also, none of the predictive formulas correlated with measured energy expenditure. Mehta et al. (6), measured REE using an in-line IC in 30 children with single-ventricle physiology after Fontan surgery, mean REE at 8 h after surgery was 57 ± 20 kcal/kg/day, also, measured REE had poor correlation with equation-estimated energy expenditure. The authors concluded that in the absence of IC, using formulas to estimate caloric requirements will increase the risk of overfeeding. Floh et al. (79), reported IC measurements in 111 children after CPB for CHD, measured REE decreased from 51 to 45 kcal/kg/day at 72 h postoperatively, indicating an increased inflammatory condition after cardiopulmonary bypass. Zhang et al. (81), reported a review of measured REE using IC in children after surgery for CHD, they concluded that daily indirect calorimetry provides the best estimate of energy needs for this population of children. Roebuck et al. (80), reported results of REE in 107 children following CPB for correction of CHD. Several formulas were compared vs. REE; Dietary Reference Intake, Harris Benedict, Schofield, and the World Health Organization. All the equations incorrectly estimated REE after surgery, with a predominance of overestimation, suggesting that IC remains the best method of estimating REE after CPB. In summary, all these studies indicate poor agreement of the use of formulas to estimate energy expenditure in children after CPB for correction of CHD, making the use of indirect calorimetry the ideal method to estimate caloric needs for this population.

### Energy expenditure in children with burns

Several studies have published measurements of energy expenditure in children with burns (43, 82–85), and exfoliative skin disorders (86, 87). Goran et al. (43), measured TEE in 15 children with burns using the doubly labeled water technique; TEE was 1.18 ± 0.17 times the REE measured by indirect calorimetry, and significant correlation among the two methods was found (*r*^2^ = 0.92, *p* = 0.002). the authors recommended that optimal nutrition support will be adjusting the REE by a factor of 1.2 in convalescent burned children. Gore et al. (82), used IC in 74 children with ≥40% total body surface area (TBSA) burns and compared to formulas. The authors concluded that formulas overestimate caloric needs in children with severe burns and that measured REE requires an additional correction factor of 30% to maintain body weight. Mayes et al. (84), measured REE in 48 children younger than 10 years of age with burns (∼ mean 30% TBSA). The study found that formulas for children with burns overpredicted energy needs and when indirect calorimetry is used in burn patients in this age group, the application of a 30% activity factor is recommended. Liusuwan et al. (83), reported values of REE in 10 children with burns (35%–97% of TBSA). The authors compared the measured REE multiplied by a factor of 1.3 with three formulas (Harris Benedict, Mayes, and World Health Organization) multiplied by a factor of 2. The results indicated that the accuracy of these formulas in predicting measured REE adjusted by a factor of 1.3 was variable and larger studies are needed to improve the prediction of energy needs in burned children. Suman et al. (85), measured REE in 91 severely burned children between 3 and 18 years of age (average of 60% TBSA). The authors compared measured REE vs. Harris Benedict, Schofield, and World Health Organization formulas without correction factor. The results showed the three formulas underestimated measured REE by indirect calorimetry with the mean percent of predicted calculated from the three equations similar on average at 152% (1,203–1,219 vs. 1,851 kcal/day from the measurement). The Bland-Altman analysis showed poor agreement between measured REE and the predicted using the three equations. The authors concluded that indirect calorimetry be used to determine REE in severely burned children. In summary, energy expenditure is elevated in children with burns and is proportional to the body surface area affected. The use of formulas to predict energy needs in this population is not accurate making indirect calorimetry the recommended method to estimate energy requirements in children with burns; this recommendation is endorsed by several scientific organizations who have published nutrition support guidelines for patients with burns (88–94).

### Use of the respiratory quotient to adjust caloric intake

Several studies have reported on the use of the RQ to adjust the caloric intake and on the metabolic evaluations in critically ill patients (22, 28–32, 95). The results of these studies have shown that indirect calorimetry is vital to modifying individual nutrition plans in patients admitted to the PICU, especially in patients with pulmonary disease, given the fact that an excess of dietary carbohydrates may result in an increase in respiratory work from an increase in VCO_2_ and make the process to wean from mechanical ventilation more difficult.

The RQ has been used as an indicator of excess or deficient caloric intake in critically ill pediatric patients with variable results (28–32). Mehta et al. (32), published results of indirect calorimetry in 14 pediatric patients admitted to the PICU. The RQ of patients classified as hypermetabolic [RMR/Basal metabolic rate (BMR) > 110%] [*n* = 7, 50.3 ± 64.2 (SD) kg] was 0.85 ± 0.03 with a caloric intake of 1,464 ± 1,008 kcal/day; and the hypometabolic patients (RMR/BMR < 90%) [*n* = 7, 44.5 ± 59 kg] had an RQ of 0.94 ± 0.06 with a caloric intake of 935 ± 559 kcal/day. The authors highlighted the importance of using indirect calorimetry to adjust individual nutritional support during the patient's PICU stay. Three clinical studies in pediatric patients have determined sensitivity and specificity values of RQ as a guide to assess for excessive or deficient caloric intake. The study by Dokken et al. (28), included 30 patients on mechanical ventilation admitted to the PICU; reported an RQ value of <0.85 as having a sensitivity and specificity of 27% and 87%, respectively; and an RQ value of >1.0, had sensitivity and specificity of 21% and 98%, respectively. The report by Hulst et al. (29), involved 95 PICU patients and reported sensitivity and specificity for an RQ value of <0.85 (deficient caloric intake), as being 63% and 89%, respectively; while the sensitivity and specificity for an RQ value of >1.0 (excess caloric intake) was 21% and 97%, respectively. Kerklaan et al. (30), studied 78 pediatric patients on mechanical ventilation and concluded that the identification of patients with excess or deficient caloric intake by way of IC and RQ values depended on the definition that was used to categorize the patients. Liusuwan et al. (31), reported on 74 pediatric patients with burns over 20% of their total body surface area with a sensitivity and specificity for an RQ value of <0.85, of 40% and 77%, respectively, and for an RQ value of >1.0, sensitivity and specificity values of 23% and 85%, respectively. Based on these studies, it can be determined that the RQ in critically ill pediatric patients has a low sensitivity and adequate specificity in identifying patients who suffer from an excess or deficient caloric intake.

In conclusion, indirect calorimetry remains the gold standard for measuring energy expenditure in critically ill patients. It is important to understand the methodology behind this technique, as well as the cost-benefit ratio to optimize its use in a critical care setting with patients who have changing metabolic needs.
